# Hospice nurses’ emotional challenges in their encounters with the dying

**DOI:** 10.3402/qhw.v11.31170

**Published:** 2016-06-01

**Authors:** Lina Paola Ingebretsen, Mette Sagbakken

**Affiliations:** Department of Nursing and Health Promotion, Faculty of Health Sciences, Oslo and Akershus University College of Applied Sciences, Oslo, Norway

**Keywords:** Nurses, emotional dimension, palliative care, professional identity, qualitative interviews

## Abstract

The purpose of this study was to explore nurses’ emotional challenges when caring for the dying in hospices. The study has a qualitative design, and knowledge was developed through a dialectical exchange between theory and data. Ten individual in-depth interviews were conducted with nurses recruited from two hospices in Denmark. Although all of the nurses said that they experienced emotional challenges or felt emotionally touched during their work, the study found a variety of opinions related to the extent to which their emotional reactions should be revealed in their role as a hospice professional. The participants described their emotional challenges as being simultaneously draining and enriching experiences leading to personal and professional growth and development. The study may contribute to increased awareness of emotional challenges for hospice nurses, which involve continuous reflection and balancing between meeting the dying as a human being and meeting the dying as a hospice professional.

Being a hospice nurse is described in research as a complex and multifaceted role with large amounts of complex emotional exposures (Broom et al., 2014; Seed & Walton, 2012). Researchers often find that hospice nurses are reluctant to talk about their own emotional experiences (Trier, 2006), and research about hospice nursing increasingly highlights the need for emotional challenges to be clarified, acknowledged, and verbalized (Bruce & Boston, 2008; Li & Arber, 2006; Melvin, 2012). Research reveals some divergence considering professionals’ emotional involvement, as most studies focus on either the difficult or the constructive elements when facing the dying (Fox, 2006; Li & Arber, 2006). Some studies point out that hospice professionals may find that witnessing the dying on a daily basis can lead to personal and professional growth and development (Bruce & Davies, 2005; Kehoe, 2006; Sinclair, 2011). However, most research focuses on the difficult aspects of professionals’ emotional exposure (Broom et al., 2014; Devik, Enmarker, & Hellzen, 2013; Harris, 2013; Melvin, 2015; Sardiwalla, VandenBerg, & Esterhuyse, 2007). Achieving emotional distance tends to be described as particularly important and as a “professional skill” to avoid long-term negative consequences for the nurses who face the dying on a daily basis (Broom et al., 2014; Fox, 2006). Contrasting research shows that hospice nurses feel they are exposed to too much emotional pressure and that they experience a lack of emotional support for themselves (Harris, 2013; Melvin, 2015). Similarly to the patients, professionals can neither control death nor know all the answers related to death itself or how the dying process will proceed for each person. Philosopher Frode Nyeng (2006) points out the complexity of emotional aspects for professionals, underlining that every person will have their own way of dealing with both emotions and death, depending on cultural and social factors. In this study we find support in Nussbaum (2001) who understands emotions as embodied and allied with thoughts and beliefs. Further, Svenaeus (2009) explains that emotions are always directed towards someone or something, which again leads to an emotional reflection that gives us an opening to understand both others and ourselves. As social norms and personal histories shape emotions, the emotional aspect within each nurse are according to Nussbaum (2001) recognized as important for the nursing practice. Nurses are a diverse group of people who work under different conditions, systems, cultures, and values. The purpose of this exploratory study is to examine nurses’ emotional challenges when caring for the dying in a hospice setting. Subsequently, we aim to gain knowledge and to contribute to verbalizing emotional challenges experienced by hospice nurses.

## Method

A qualitative design was used to explore a range of emotional experiences in caring for the dying. The process of developing knowledge has been structured as a constant circular and dialectic movement between theory and data. According to Fangen (2010), this dynamic procedure is described as an abductive approach, in which theoretical insights are discovered from the data in conjunction with a primary theoretical framework. This study was not framed by any definite theory that was to elucidate our material ahead of the data production. We were, however, influenced by the simultaneity paradigm as a philosophical assumption (Barrett, 2002; Parse, 1992). According to Parse (1992) there exists two major worldviews within nursing science, the totality and simultaneity paradigm. The totality paradigm consists of ideas portraying a traditional nursing practice, where the goal is seen as curative, the main focus is on problem-solving, and the human is seen separate from a changing environment. The simultaneity paradigm is, on the contrary, rooted in the worldview of human as unitary, both in the sense of being unitary with its environment, but also in the meaning that you cannot understand a human by studying only the parts. Thus, the simultaneity paradigm focuses on the lived experience of each human whereas the main goal is to uncover the subjective understanding of health and well-being (Barrett, 2002; Parse, 1992). The simultaneity paradigm has inspired the overall understanding of the material and represented a source of reference when seeking to identify relevant theories during the analysis.

Our research method is inspired both by hermeneutics and phenomenology; and represent a combination of both; a phenomenological hermeneutical approach (Lindseth & Norberg, 2004). According to Malterud (2011), phenomenology focuses on the human experience while hermeneutics is based on text interpretation. Lived experience is, as explained by Lindseth and Norberg (2004), an expression that refers to the phenomenological approach and in this study emotional challenges represents the phenomenon we aim to explore. As the interviews provided expressions related to nurses’ emotional challenges, we intended to explore the essential meaning of their descriptions. We adopted Lindseth and Norbergs (2004) interpretation as they argue that one should strive to understand phenomenon's as they are experienced and described by the participants of the study. However, for analytical purposes, the participants lived experiences has to be adjusted into a fixed text, and for that reason the hermeneutical approach can be seen as a mandatory part of the qualitative research process. Although the hermeneutic method is established in regards to written interpretation of completed texts, the researchers contribute in producing the textual material (Lindseth & Norberg, 2004). Furthermore this study is inspired by hermeneutics, as we have obtained a constant and dynamic dialogue with the material; moving between perceiving and analysing the material at a detailed level (single interviews) and by trying to understand how the single pieces fit into the entire picture. To optimize the analytical process and to achieve a systematic approach to the data material, we worked with each interview immediately after the interviewing session. Directly after each interview the researcher wrote down her first impression and reflections upon the interview. Before a new interview was conducted, the recording were listened to and transcribed, followed by a more detailed reflection-log, consisting of descriptive and analytical notes. Based on experiences, reflections and identification of novel topics that emerged from the single interviews, adjustments of the interviewing guide were constantly made. When initiating the coding process, the researchers again started by focusing on each interview (the parts), but now from another angle, as we had gained new insight after transcribing and reading all of the interviews (the whole). According to Kvale and Brinkmann (2010), this constant movement between single parts and the whole is required to bring the participants’ expressions to a higher level of understanding.

### Setting

Two hospices within the special health-care service in Denmark were chosen as contexts for data production. One is situated close to a large city, while the other is located in a rural district. By selecting two hospices in different locales, we aimed to include any possible differences caused by their contexts. Denmark has 18 hospices across the country, with the first one established in 1992 (Palliativt Videncenter, 2015). Norway has only one hospice in the special health-care service, established in 1994 (Hospice Lovisenberg, 2010). Another motivation for choosing Denmark as a field was that Hospiceforum Norway states that one of their main goals is to establish more hospices in Norway, inspired by the Danish model (Hospiceforum Norge, 2011). One of the authors was an employee at the Norwegian hospice in the special health-care service and has a special interest in the field. Using the Norwegian hospice as a context for the study would hence lead to different sorts of challenges, such as a greater risk of not noticing new and central elements. By selecting Denmark as a field, we intended to improve our ability to view a well-known context as being new and unfamiliar by distancing ourselves from an already-known research field (Thornquist, 2003).

### Participants

The data is based on 10 individual in-depth interviews with nurses working at the two hospices. Our intention was to acquire information-rich material about nurses’ emotional challenges in facing the dying, and we sought to achieve “maximum variation” in our purposeful sampling strategy (Palinkas et al., 2015). By striving for “maximum variation” in our sampling, we sought to identify central themes and core experiences, but also unique aspects emerging from a sample of variations. This approach can increase the study's validity and also enhance and broaden understanding, through nuances and details related to the theme of the study (Malterud, 2011). Several approaches were used to achieve diversity in our sampling. The two Danish hospices included in the study were located in different surroundings. Six participants were recruited from one hospice and four from the other. The participants were between 20 and 60 years old. They were all fulltime employees and female. Our goal of “maximum variation” in our sampling was partly achieved, although we did not recruit any men in the study. We were informed that the hospices did not have any fulltime male employees. According to Thoresen (2008), few men work as nurses in hospices and generally there are more female employees within the hospice field as a whole. As we collected material that revealed several perspectives and that provided many nuances, we considered that saturation was reached after in-depth interviews of 10 participants (Malterud, 2011).

### Data collection

The management at each hospice assisted with recruiting participants following our selected inclusion criteria. All interviews were completed by the first author and lasted 60–90 min. An interview guide was developed and used to provide structure and focus on topics related to nurses’ emotional experiences. The topics in the guide were developed from the research literature, theoretical perspectives, and the research questions. A relaxed structure prevailed during the interviews. Participants were given the space to tell their stories openly, expand upon their experiences, and thus deviate from the interview guide. The participants had the opportunity to select the place for the interviews and they all chose a conversation room at their workplace. The researcher introduced each interview with small talk with the respondent with the purpose of establishing a relaxed and natural atmosphere. The researcher then introduced herself and repeated the aim of the study to ensure that participants were well informed about the study's topic and to achieve a comfortable environment for verbalizing emotional challenges. A characteristic of the interviews was that they proceeded as a dialectic interaction between the participant and the researcher. The progress of the conversation was different in each interview, and topics from the interview guide were introduced in different orders. The researcher experienced that some participants were confiding in her to a deeper level in their reflections about topics than first expected. In such situations, the researcher chose to follow the respondent without interruptions or limitations. Hence, we did not necessarily discuss all the topics listed in the interview guide, but some themes were explored more deeply and new topics were included. After each interview, both authors set time aside to discuss preliminary findings, reflect on the themes and the questions that were discussed, and subsequently use our expanded understanding to adjust our interview guide as well as our general approach to the topics and the participants. All interviews were audio tape-recorded and transcribed verbatim.

### Ethical considerations

Participants received a written informed consent form, which they signed and returned to us in advance of being included in the study. The participants were informed that their participation was voluntary, that they had the right to terminate their participation without giving a reason. They were also informed, that the participants’ quotes would be anonymized and that personal details such as age would not be presented together with the quotes, to prevent identification. To ensure that they had understood the given information about their involvement in the study, information from the informed consent was repeated before the interview started. The Norwegian Social Science Data Services (Norsk Samfunnsvitenskapelig Datatjeneste, 2011) granted permission for the study (No. 28042). Examining emotional challenges facing the dying was expected to involve confrontation with adverse feelings for the participants, although research findings also demonstrate that participants find that telling stories about their emotional challenges can be experienced as useful (Cain, 2012). As the researchers did not know how participants would experience their participation in the study, we provided an option for a second interview. The intention with this option was to give participants an opportunity to clarify potential misunderstandings or questions that might have been revealed because of their participation. None of the participants terminated their interviews nor requested a second interview.

### Data analysis

Data were analysed based on Malterud's (2011) “systematic text condensation,” which is a modified version of Giorgi's (1985) “phenomenological analysis.” The analysis was not seen as an isolated process, but rather as a continuum starting from the beginning of the study until its completion. To optimize the analytical process and to maintain a systematic approach to the material, the first author wrote a reflection log concentrating on the overall impression of each interview. This log was written once each interview was finished, without listening to the recording. Reflections included a focus on the context of the interview, the structure of the interview, and what worked well or could be improved. The second reflection log was more specific and followed listening to the interview record once. This log was centred on the thematic content of the interviews and inspired reflections on our understanding of the different themes and subthemes under discussion. In this log, we also focused on the role of the researcher. We reflected on how the researcher experienced the interview and how the researcher influenced the interview situation and consequently the outcome of the interview. Reflections upon the principal researcher's influence on the interview situation were mainly related to the potentially sensitive focus of the study. Based on knowledge from earlier studies, in which demonstrate that hospice nurses often are reluctant to talk about own emotional experiences, we expected that there would be a somehow difficult topic to explore. The log reflected the researchers’ distress of stepping too close in on the respondents’ emotional life. By asking the nurses to describe aspects of their professionality in which they experienced as challenging or difficult, the principal researcher was concerned about invading the nurses’ emotions and thus confirm potentially negative impressions the nurses have of themselves. Reflections were also related to the principal researchers own experience from working in a hospice, and how this mutual experience with the participants either possibly would work as an door opener for the participants to elaborate their descriptions, or perhaps as an barrier, in which could lead the nurses to present themselves as more skilled and infallible than they actually experienced. Instead of ignoring the influence, the researchers have continuously tried to identify and understand the potential impact we have had on the material. After the two logs were completed, the transcription of the interviews began. All transcriptions were made by the first author. Thus, the transcription process also opened up new analytical reflections and constituted a part of the analytical process. To achieve an overall impression of the material, both the first and the second author read all the texts as a whole, focusing on identifying preliminary themes. Step two involved breaking the structure of the interviews by splitting the material into relevant categories describing different emotional experiences (Malterud, 2011). By decontextualizing the text, the researchers discovered how fragments of the text could support and strengthen related segments. Although Malterud's (2011) steps were used to analyse the data, step three was slightly modified by the researchers. After identifying subgroups of the relevant material, Malterud (2011) describes the process of creating condensed statements or citations based on participants’ expressions. We preferred to maintain focus on the participants’ own language, and identified subthemes of the material by color-coding participants’ expressions. Finally, we summarized the material by linking the thematic blocks with the subgroups to acquire renewed understanding of the material and its content and to articulate a coherent presentation of the material. The original transcriptions were reread to ensure that the written presentation was anchored in the original context. Quotes from the interviews are used to exemplify and illustrate the renewed understanding of the material.

## Results

The research questions guiding this study focus on the emotional challenges experienced by nurses when facing the dying in a hospice context. Four themes emerged from the qualitative analysis of the data. One theme is centred on nurses feeling emotionally touched. Another theme related to the nurses identifying themselves with the patients’ situation, and accordingly needing to distance themselves. A third theme relates to the emotional challenge in balancing personal and professional dimensions when facing a dying patient. The final theme illustrates how closeness to death also functions as a reminder of the nurse's own mortality, with implications for the nurse's perception of her own life and death. In general, emotional challenges were related to dynamic movements between both enriching and draining experiences.

### Emotionally touched

All participants said that they felt emotionally touched in different ways by the dying patients. Through recognizing their own emotions when facing patients, some nurses described how they had the ability to suppress or hide their emotions for a while and to reveal them later at a more convenient time. Appropriate times for revealing emotions were described as individual conversations with colleagues or at home with spouses. Although some nurses said they could hide emotions, all participants emphasized the importance of acknowledging their emotional reactions. Not being frightened of emotional involvement was emphasized as a crucial capacity. Allowing themselves to be emotionally touched by patients also required the nurses to be cautious, so that the emotions would not become overwhelming. Many explained how they were continually balancing between the patients’ and their own feelings, and expressed a concern of patients’ emotions “occupying the room meant for their own feelings,” as stated by one participant. One nurse described how emotional experiences could be enriching and draining at the same time and why finding a balance was important:

If I felt on a daily basis after leaving work, I did not have room for anything else, not enough space for other feelings, or if I had forgotten what is really most important for any human being … […] exactly who I am as a person, then it (the emotional challenge) would not be acceptable. Yet it is fantastic meeting other people, and always experiencing emotions in such a big spectrum. This I find enriching.

Being emotionally touched was frequently related to “witnessing suffering.” Witnessing or “partaking” in suffering was often associated with a distinction of doing something “for” patients, versus being “with” patients and their caregivers. A nurse described how she perceived her presence as emotionally challenging when she felt she could not do anything “for” the patient and relatives:

It was difficult being there with them. In one way, you really want to do something but there is nothing to do. You cannot take away their pain; you cannot relieve them from their suffering. One must only be there together with them. In a room filled with pain and sadness.

Being “with” patients, but not having the ability to change the patients’ life situation, was underlined as an emotional challenge. Strong words and expressions were used by nurses in describing this challenge. One nurse verbalized how she felt:

It hurts to feel that I am inadequate, that I cannot do anything further […] one might call it powerlessness, or call it inadequacy. But when I recognize that I cannot solve a life problem, I have to leave it there. […] Yet it does not prevent my feeling that it would be wonderful to have the ability to help.

In contrast to this nurse, another nurse illustrated that she had developed increasing confidence in being “with” patients. This nurse specified that she preferred that “the moment” should define how to interact, although she was still disturbed by thoughts on how she ideally ought to appear:

A young woman asked me if I thought she was about to die at that very moment. […]

I thought, what I am supposed to say now, what have I learned are the right words to say? Then I assumed there is nothing to say, other than what do you think? And she said, “I believe so.” It was actually true; she died while I held her. […] It was the right thing to do, not saying all I have learned as the ideal things to say, but rather rest in the moment. […] Having the courage to be present in the moment without acting in any way.

The participant described this moment as one of the most challenging situations she had ever experienced as a nurse because of being fully present with the dying person without “being caught by panic,” or considering what would have been the most “professional” action. While several nurses described emotional challenges as somehow draining experiences, others described such challenges as both enriching and important experiences in the framework of providing care. A nurse elaborated:

For me, it is also important that we are letting ourselves be emotionally touched and that we sense the powerlessness. If I sense this much [illustrates about one centimeter distance between her fingers], then it gives me just a tiny little impression on how the dying and their relatives are experiencing their situation. Therefore this little sensation assists us not to be emotionless with those we are facing.

The nurse quoted above explains that although she was able to sense only a small part of how patients and relatives were experiencing their situation, this awareness gave her the possibility of increasing her understanding of their experiences.

### Identifying and distancing

The nurses told that they experienced both “identifying” and “distancing” as emotional reactions when facing the dying person. Nurses both identified themselves with or strived to make a distance from the dying and their relatives. When talking about experiences related to distancing and identifying themselves, a dynamic connection between these terms was revealed, as distancing was sometimes explained as a reaction to their identification with the suffering person. Whilst distancing could be described as a consequence of nurses not being able to absorb more suffering, distancing was also described as helpful in a prolonged process of relating to the suffering. A nurse exemplified this mechanism by describing her need for distancing as she identified herself with a patient's daughter:

I remember I thought I really had to keep calm. I was the one who needed to be balanced. One of the daughters was really emotional. Screaming, crying, and very intense in her response. […] It all came so suddenly […] I was totally unprepared to be suddenly confronted with such strong feelings, because it was a very powerful reaction. […] But I think I would have reacted the same way if it was my mother.

The nurse identified herself with her patient's daughter and thought she would have reacted the same way in a similar situation. This identification, in combination with the daughter's strong emotional expressions, made her feel a particular need to distance herself from the situation. As a consequence of identifying themselves too intensely, several nurses said they had developed different strategies to gain distance. Having access to such strategies was thus seen as required when facing the dying and their caregivers. One nurse said she had named her strategy and called it her “psychological support.” She repeatedly said to herself “this is not my family, this is not me.” This same participant said that she was aware when this strategy was necessary because she recognized situations that affected her on a deeper level.

When explaining distancing strategies, many nurses used different metaphors illustrating how they were exposing themselves to different extents. “Entering into a shell” was a metaphor often used. Participants described that the shell provided the opportunity to remove their constructed barrier to patients’ suffering, consciously, layer by layer. Thus, the nurses could decide to some extent on what level they were involving their personal dimension and allow themselves to be emotionally touched. A nurse explained how she exposes herself at different levels:

We are being confronted with some conditions in patients’ lives and deaths that engage something deeply within [points at her solar plexus], existentially, and we are affected by this. […] I think I am affected at my existential level, as a human being. When the nurse is peeled off, when the daughter is peeled off … then it is me as human, on the deepest level that is affected, my existence.

In addition to the metaphor of “shell,” expressions such as “wearing a coat,” “taking care of oneself,” and “technical busyness” were used to describe different detachment strategies. “Technical busyness” was described as a “hard” way of achieving distance and this strategy involved making oneself unreachable:

You do not ask [the patient or relatives] about severe things, because you really do not want to talk about it and because you cannot stand it yourself. […] instead, you act. It is a shell, a professional shell that warns everyone; I am busy, I am handling this. So the wife by the bedside, she absolutely does not dare to ask any questions as it is very important what the nurse is doing […] one is signaling technical busyness. A person with authority [emphasizes the word authority].

In contrast to “technical busyness,” the strategy of “wearing a coat” was explained as a milder way of distancing oneself. With this strategy, the nurse's role was characterized by much gentleness and compassion. This nurse appears as the one who is always in possession of excessive friendliness; she avoids any complications and has only superficial communication and interactions with patients. With this approach, the nurse signals that she does not want to face any difficulties or challenges, and this strategy was subsequently also described as a way of achieving distance from the suffering person:

They [nurses] […] are not touching anything severe, there is no dynamic. […] It is just the status quo. This cute little nurse, she who holds a cloth to your forehead […] it should also be a part of the nursing, but not always, because then it becomes a coat. […] if you want to influence something, if you want the art in nursing, this coat has to go.

Even though many nurses criticized the distancing strategies, the need for distance was also recognized as mandatory to uphold their role as supportive and safe professionals. The nurse quoted below described how working with her own emotional reactions, and subsequently balancing between distance and identification, was a requirement for her being able to face her patients’ suffering:

I did not know how to face it, since I found it hard. He often told me; I am not afraid to die, I just do not want to. […] Next time he told me the same, I said to him; I understand, I would not want to die and leave my children […] It was difficult and I was exhausted when I went home, because it really affected my feelings; he was a father leaving his children. It was hard, it really was. And I think the fact that we were at the same age, also played a role […] I shed many tears, I surely was a bit afraid to face it as I was afraid of breaking down or that I would not manage to stay there, with him. But I established enough courage to face him. Afterwards I am glad I did …

Balancing identifying and distancing can be understood as a dilemma in the nurse's interaction with the patient. The nurse quoted above explained that her identification with the patient was the reason why she had difficulties in facing his suffering and at the same time the reason for understanding him. The quote exemplifies how distancing strategies might function as preparing strategies when more time is needed to be able to face the suffering.

### Person and profession

All participants stressed that hospice work required a balance between their personal and professional dimensions when facing patients and their relatives. Distinguishing their person fully from their professional appearance was explained as impossible. The nurse quoted below exemplifies how she perceives involvement of both her personal and professional identity while working at hospice:

The person you are at work influence how you are, as a person. All the personal characteristics you have, but also your professional reflections. I actually think these things belong together. I do not think you can separate it. You will bring something from your own life that activates how you are being affected here. So you are using yourself a lot.

Although all the nurses agreed they could not fully separate their person from their professional performance, there were several opinions on how to achieve the best balance. One participant said she felt enriched on “two sides,” explaining the twofold enhancement as a consequence of letting herself be involved with both her professional and personal identity:

Working in hospice is an enriching experience. It enriches me a lot as a human and it enriches me as a professional nurse. For me it is a dual enrichment. My professional identity and who I am as a person is strongly incorporated and unified. That is why I think I am being enriched in both dimensions, as a human and as a professional.

This nurse is explaining the duality of being enriched, based on her strong interconnection between her personal and professional identities. Several nurses shared her opinion, while other participants disagreed and strived to separate their personal and professional appearances. A nurse describes how she tries to keep the two “worlds” apart:

My personal world is separated from my professional world. […] I think it is important that when I leave work, I manage to open up for something else, my private life. […] This does not mean that when I am home I do not think of our patients. If so, I would have been inhuman. […] But generally, I am trying to keep those worlds apart.

At the same time as she tries to keep the two “worlds” apart, she also acknowledges the dynamics between the personal and professional dimensions. Many participants said that the ideal of keeping the personal and professional aspects apart emerged from expectations of their primary understanding of what constituted being professional. Participants said that such expectations were both verbalized and heard from others, but also incorporated as anticipations within themselves. Many nurses had experiences with colleagues that had to quit hospice work and they linked this to unrealistic expectations on how to be a hospice nurse. A nurse elaborates:

Because we do all have expectations of what constitutes a hospice nurse. She has plenty of time, she will listen to everything patients are telling her, she will always know the right things to say, and she will always act correctly. Altogether you will forget that she is also a human, making mistakes and saying the wrong things.

The expectations involves a risk about being too anxious about completing the role as “professional,” hence the nurse might forget that she is also a human who sometimes makes mistakes and does not know what to say. At the same time, the above citation also illustrates nurses’ different perceptions and understanding in regards to their role as a professional, and what constitutes this role. For some nurses it was necessary to talk with their close ones about challenging experiences from work. One informant had such a huge need for debriefing difficult situations that she wanted “education” for her spouse on how to talk about her work experiences:

I would appreciate if my spouse were taught about what we face here, the psychological aspects we are dealing with. […] this would provide a broader understanding of my work. […] Sometimes it would be great to talk to someone at home who understood, a person not involved in the situation.

Most participants stressed the need for time and opportunity to talk about complicated situations during work hours. Several nurses emphasized the fellowship with colleagues as vital in managing and dealing with difficult situations. To achieve an opportunity for an ongoing dialogue among the colleagues, nurses desired a physical “room” just for them as employees, and also time in their daily schedule to allow conversations to develop:

I miss more time just for us as colleagues, where we would have the opportunity to talk freely. We are actually always together with the patients. That is also nice, but […] it would be good if also we had some time only for us. Talking together and letting the conversation flow, as something surely would be brought up.

Through acknowledgment of emotional reactions, many participants described that they felt they were being recognized as professionals. Frequent conversations with colleagues were emphasized as important for the nurses’ well-being and satisfaction related to their work.

### Closeness to death—a reminder of life and death

As a consequence of constantly working close to death and the dying, several nurses said they had learned to accept death as a natural element of life. Some of the nurses explained they felt honoured because of their opportunity to spend time with people who were living their last part of life, as the experience was perceived as both personally and professionally enriching. Although participants expressed honour, enrichment, and gratefulness, a few nurses also said they perceived the closeness to death as intense and challenging. One participant explained this duality:

Even though I think it is very life-confirming working here, it can certainly be difficult. It is also nice getting inside and becoming aware of oneself. Noticing that one can be touched on a deeper level. Somehow, I believe it is really intense. […] and I can use the experience also in my personal life.

This participant emphasized the work as enriching in her own life, but also as an experience that was challenging and intense because closeness to death and the dying was touching something profound within herself. Although closeness to death led to increased awareness of the nurse's own mortality, all participants stressed that this familiarity had been most important in regards to awareness of life. In contrast to what several of the nurses had expected, the hospice environment was dominated by joy in life and cheerfulness. A nurse explained how she had experienced a focus on life as more central than she first expected when starting hospice work:

Even if it sounds odd, hospice is actually a very life-confirming place. […] It is more centered on life than I expected. Of course, it is also about death, but that is not the most important thing. And it may sound strange for those who are not familiar with the hospice field. […] It is an incredibly lively place to be.

The nurse described the hospice as a lively place and used the expression “life-confirming,” an expression repeatedly used by the participants when talking about their work. Many nurses expressed that constantly being confronted with death and their own mortality had an impact in their own lives. They experienced the work as “life-confirming,” and that their lives had become more intense than they were before they started hospice work. One participant explained how she experienced reaching a new dimension in her life:

Being fortunate to observe the world through the eyes of a person who will soon be gone. […] that makes life more intense. […] some of the experiences patients bring up, when they see things I never would have noticed. Suddenly I am seeing a new dimension of myself; it provides me with a lot. Because I am privileged to see the world with eyes that are not mine. With eyes observing so intensely, only like you are able to when you know that you are soon going to leave.

When questioning what it meant that life had become more intense, many participants said that they were reminded daily of how fast life could change, they had achieved increased awareness on how important it was to enjoy their time and enjoy life. Many of the participants said they no longer deferred things they desired to do in their own life, and emphasized that closeness to death had led to greater consciousness of how they wanted to live their lives and what they perceived as important. A nurse describes how facing death and the dying had become a reminder on how to live her life:

You think about how rapidly things may change. From being fine and healthy, as you hopefully will be. But we see younger people coming here, and this becomes a reminder about living life, take care of the small things. One should not think; let us defer this to later. One should try to focus on being present. […] Because suddenly it is too late.

While many of the participants said that they had achieved positive dimensions in their own life as a consequence of their closeness to death, this enrichment was also experienced as somehow difficult. This ambivalent feeling was linked to the fact that they had achieved insight into their own life at the expense of their patients’ suffering. Thus, other people's suffering was perceived as encouraging an increased awareness of how the nurses lived their own lives.

## Discussion

The findings in this study illustrate that nurses working in hospices experience emotional challenges from their exposure to suffering and death and their increased awareness of their own future mortality. Different situations, involving different exposures and emotions, brought about dynamic movements between identifying with and separating themselves from the suffering of patients and their relatives. Through preconceptions, demands, and expectations of what being professional implies, the nurses’ perception of their own professionalism was challenged. The nurses were also emotionally challenged by their experience of being enriched in their own lives as a consequence of facing the dying.

### Emotional challenges explained by guilt

Many participants said that they felt enriched as a result of facing people who were living in their last phase of life. This finding is similar to findings from a study of hospice nurses managing workplace stress, as those nurses also described working with the dying as an honour, as life affirming, and as encouraging them to appreciate their own lives more fully (Harris, 2013). Another study that examined qualities, characteristics, and challenges of hospice nurses in the United States additionally found that the nurses, as a consequence of facing the dying, felt they were receiving a gift of personal development that supported them in their personal life journey (Kehoe, 2006). In contrast to this research, participants in our study experienced the enrichment as multifaceted, because the patients’ suffering was seen as a requirement for the enhancement in the nurses’ own lives to happen. The Danish social observer and psychotherapist Susanne Bang (2003) have stated that facing other people's loss and traumas is tougher for professional workers than earlier expected. When witnessing others who suffer, Bang (2003) explains that one is at risk of feeling “survival guilt,” a symptom described as central for the secondarily traumatized person. Similarly, associate professor and nurse Anne Bruce and professor and nurse Patricia Boston (2008) found that the emotional impacts for hospice professionals were associated with feelings like guilt and dissatisfaction, which were often rooted in unrealistic expectations of providing the optimal care for the dying. The desire to achieve distance from the situation might be seen as a reaction to the feeling of guilt and to witnessing the suffering. A study of daily challenges faced by hospice nurses supports this view, as the authors stress that nurses must mentally disengage themselves from the patients to maintain their own mental stability and well-being (Seed & Walton, 2012).

Canadian Irene Renzenbrink (2005), seen as a prime pedagogue and supervisor in dealing with grief and loss for professionals within the palliative field, highlights the need for professional supervision and staff support as a mandatory requirement for professionals in accomplishing their work facing death and dying. Participants in this study were of the same opinion, as they underlined the importance of professional supervision. Although participants said they were receiving professional supervision to some extent, they emphasized their collegiate community as more important to be able to reflect on different situations and feelings. This experience is supported in several studies as findings highlight the need for organizational facilitation to provide opportunities for open discussions and emotional debriefing with fellow nurses (Bruce & Boston, 2008; Harris, 2013; Malloy, Thrane, Winston, Virani, & Kelly, 2013; Seed & Walton, 2012). Furthermore, Bang (2003) states that the feeling of survival guilt can be avoided and that professional helpers might continue their compassionate work without being emotionally drained. However, professional supervision is underlined as a requirement for this to happen. By acknowledging the call for sufficient staff support, Renzenbrink (2005) declares that professionals will achieve both personal and professional growth and development.

### Emotional challenges explained by role

Goffman's (1992) role theory can be used to interpret parts of the empirical material in this study. Goffman uses the imagery of the theatre to portray important characteristics of human social interaction. His explanation is that everyone who is involved in the interaction will be positioning themselves based on expectations and impressions gained in the very first meeting. Many participants said that they often faced expectations associated with the role of being a professional nurse. Expectations could be spoken or unspoken, as well as derived from themselves and others. Several participants explained how they used different strategies to control patients’ and caregivers’ impressions of themselves. Goffman (1992) further underline controlling emotional expressions as crucial, and as the masterwork in demonstrating the extent to which nurses are able to complete their acting roles. One strategy the nurses described that clarifies this argument is the strategy illustrated as “technical busyness.” By using this strategy, the nurses wanted to signal busyness and authority to the surroundings, and as a result express unavailability and a desire to be left alone. Other participants explained how they covered themselves in a “coat” or kept a “shell” to maintain their performing role as professional nurses. Many nurses said they were always with patients and caregivers, and this can be understood as they were always performing in “front of the stage”; continuously controlling emotional reactions, on stage, in front of the audience, acting their role. However, Goffman (1992) points out that the actors also need a room behind the stage. Some nurses said they used their family to talk about challenging situations at work, which can be understood as the nurses constructing a “backstage” space at home, although it was evident that the nurses wanted to locate their “backstage” at work, with close proximity to the stage. This finding is supported in other studies, where talking about emotional challenges with colleagues was described as most helpful for hospice nurses when coping with stress (Harris, 2013). Although Goffman (1992) outlined achieving emotional distance as mandatory to achieve good performance, contrarily, the nurses said that emotionally distancing alone was not sufficient to complete their professional role as nurses. The nurses also emphasized emotional identification as important for fulfilling their professional role. Goffman (1992) underlines that an advanced actor must never let herself be emotionally touched by her own play, but rather be able to move freely between private and official areas at any time without losing her rationale. This description seems contradictory to what many of the nurses emphasized when they were explaining how working with their own emotional reactions was seen as a strength and necessary for upholding their professional role. Research about life journeys of hospice nurses supports the participants’ opinions, as nurses’ experiences of their own emotional pain were described as mandatory for helping others through their pain (Gaydos, 2004). By allowing the personal part of the identity to be part of their professional role, the nurses seem to feel stronger and more comfortable in their role as professionals.

### Emotional challenges explained by professionalism

Associate Professor at the Center for Professional Studies, Anders Molander, and Professor in Professional Studies, Lars Inge Terum (2008), point out that the main chore of health-related professionalism today is to provide a change in health conditions for the sick. This vision might represent an emotional challenge for nurses working with dying people, as the hospice work does not have a curative intention. Several participants said they had often experienced feelings like powerlessness and unproductiveness when facing the dying. Such feelings were often associated with the lack of opportunity to improve the health condition of the dying. The nurses’ descriptions of the challenges that lie in being just present with the patients can be viewed as an expression of values in the society as a whole. Nurse and professor Kristian Larsen (2007) state that the society of today, including the health-care system, is fixated by measuring and evaluating the effect of concrete procedures. Nurse and associate professor Mary Kalfoss (2001) supports Larsen (2007) as she emphasizes that the central values of today's society are more or less solely characterized by productivity and development. Being with a patient is difficult to measure; it does not necessarily require the performance of specific procedures, and will tend not to be interpreted within the frame of “production.”

Another topic that emerges from this material is the relationship between the professional and the patient. Through perceiving the professional as an expert, the asymmetric relation between nurse and patient becomes highly visible (Molander & Terum, 2008). The nurse encounters patients and their relatives through her role as a helper, while patients are in need of the help that can be provided. Professor of systematic theology and diaconal research, Trygve Wyller (2005) underlines the importance of meeting patients not only as patients, but also as people. This can be seen as a paradox; the encounter between nurse and patient has initially occurred only because the patient needs the nurses’ professional competence. Wyller (2005) underlines that this paradox might be experienced as one of the biggest challenges for the professional, as being able to encounter the patient as a fellow human being requires the professional to develop an “unprofessional judgment.” Wyller (2005) describes the unprofessional judgment as part of everyone's lives and experiences, which includes attitudes and actions we do as a result of being human. In other words, Wyller (2005) highlights the significance of personal attributes in professionals and claims that the professional role will always be affected by professionals’ personal dimension. In a case study focusing on how the lives of professionals are influenced by patients’ situations, the authors supported Wyller (2005), emphasizing that nurses must remember that at the end of the day they are vulnerable human beings, not just “professionals” (Chiera-Lyle & Arshinoff, 2015). A meta-synthesis of qualitative studies focusing on hospice nurses supports this view, as findings show that the most commonly recognized quality among hospice nurses is to be humanly present, with personal involvement seen as essential (Kehoe, 2006).

Being humanly present can furthermore be associated with nurse and professor Rosemarie Parse's theory of human becoming, as “true presence” is described as the main core of nursing practice (Parse, 1992). Parse's theory emerges from the simultaneity paradigm and the goal of the nursing practice is to improve health without considering any problems to be solved, but rather to discover wellness thru reflexive awareness and inner exploration. Reflection work between patient and the nurse is seen as mandatory for the improvement of health or well-being to occur. Or, as emphasized by Parse, it is the subjective understanding of health that is to be improved, not necessarily the health condition itself. Moreover, a shift in the subjective understanding of health may be experienced of both the patient and the nurse, as they often influence each other's reflections and views related to health and well-being (Barrett, 2002; Hansen-Ketchum, 2004; Parse, 1992). Participants in this study equally communicated that they had experienced increased awareness and new insight in their own life as a consequence of their encounters with the dying. In a study of Parse's theory in practice, the author highlights that the journey towards improvement of health and well-being will not take place in isolation, and nurses who affects and are getting affected by the process are described as privileged (Hansen-Ketchum, 2004). In a study from Canada, aiming at comparing palliative nursing practice with Parse's theory, several congruent elements were found. Focusing on the interhuman connection, where the nurse is being fully present and attentive to the patient, is outlined as one of the most consistent elements in regards to health and well-being. Other congruent elements were: understanding the whole patient, not reducing him or her to parts, and the importance of focusing on the subjective experience of quality of life (Hutchings, 2002).

Many participants in our study said they had expected hospice nurses to be “experts” and to master any situation. However, several had reconsidered their role, and now considered their professional role to be interwoven with their personal role; however, they offered varied perceptions related to the extent to which they let their personal role influence their professional role. A study that explored nurses’ experience of professional fulfilment in palliative care showed that nurses gain fulfilment in their professional role when they care for patients using creative and unexpected approaches that they would never have learned from a nursing textbook (Perry, 2009). In our study, some participants struggled to separate their personal from their professional role by trying to achieve an emotional distance from patients. Others associated the professional role with compassionate presence and emotional engagement. Many participants said that they did not always act out of expectations of the most professional approach, but rather acted out of their own intuition in the current situation. This approach to practical nursing is supported in a meta-synthesis of qualitative studies focusing on hospice nurses (Kehoe, 2006), in which the human body was used as a metaphor to illustrate the relationships among findings. Being able to confront the unknown and to rely on experience rather than formal education is emphasized as important, and a sense being located in the belly (Kehoe, 2006). Similar reflections were described by participants in this study, as emotionally challenging experiences were described as situations involving both the nurse's personal and professional experience and dimension.

As a response to the emotional exposure, the nurses emphasized that they worked on controlling their own emotional reactions to sustain their professional role. However, they were also recognizing their own emotional involvement while treating the dying. When acknowledging the emotional challenges as multifaceted and dynamic experiences, the nurses discovered the opportunity to gain enrichment and development at both personal and professional levels. This can be seen in the light of Wyller's (2005) statement that emotional experiences or feelings not reflected upon do not bring about new knowledge, and may rather lead to emotional burnout or exhaustion. Emotional experiences that are reflected upon, on the other hand, may create new knowledge and be enriching and developing, without leading to the same type of emotional exhaustion. In other words, part of the explanation why some experience some emotional challenges as draining while others experience them as enriching could be because of the level of reflection—or the possibility of reflecting—on emotionally difficult situations.

Although this study contributes rich insights into nurses’ experiences of emotional challenges in hospice, further work is needed to explore the emotional challenges for nurses facing the dying within different contexts and diverse cultures. In future studies it would be interesting to focus on different cultural dimensions, e.g., comparing experiences of nurses’ who belong to more individual oriented cultures versus more collectivistic oriented cultures. It could also be interesting to conduct research on emotional challenges that includes spiritual or religious dimensions, for instance exploring nurses’ experiences from a non-religious versus different religious believes. The same study could also be conducted within diverse contexts of the health system, including nursing homes and various hospital departments, to explore how different contexts with different aims (curative aim) may influence emotional challenges.
